# Supplementary material for: Identification of photoperiod-induced specific miRNAs in the adrenal glands of Sunite sheep (Ovis aries)
Source: Front Vet Sci. 2022 Jul 22;9:888207. doi: 10.3389/fvets.2022.888207 (PMC9354845; doi:10.3389/fvets.2022.888207)
Supplement: Supplementary file 1 [file Data_Sheet_1.ZIP › Supplementary file/miRDeep2 file.zip!/miRDeep2 file/Know_Structure/SP42A3/oar-mir-10a.pdf]

miRBase precursor : oar-mir-10a
Total read count : 173924
oar-miR-10a read count : 173014
remaining reads : 910

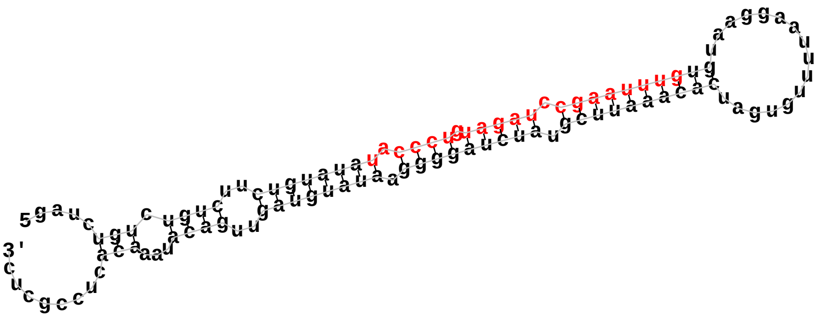

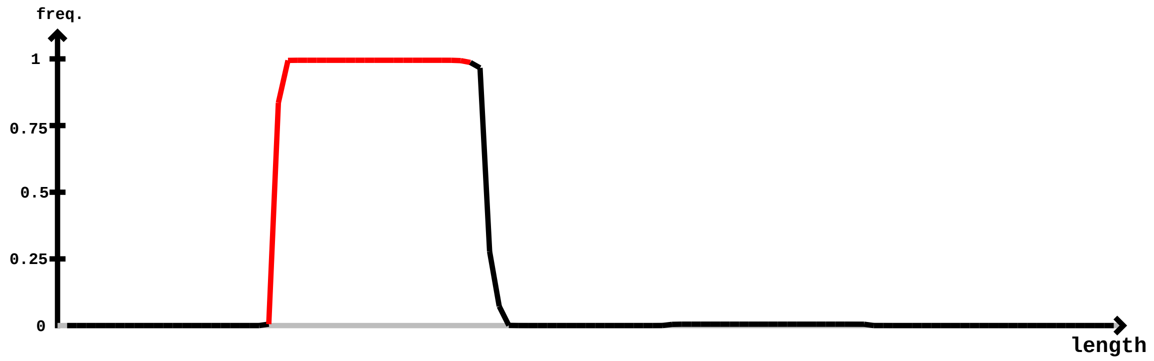

oar-miR-10a

5'- gaucugucugucuucuguauauacccuguagauccgaauuuguguaaggaauuuugugaucacaaauucguaucuaggggaauauguaguugacauaaacacuccgcuc -3'	exp		
....(((.((((..((((((((.((((.(((((.((((((((((................)))))))))).))))))))).))))))))..))))...)))........	reads	mm	sample
.aucugucugucuucuguau...	1	0	seq
.aucugucugucuucuguaua..	1	0	seq
..ucugucugucuucuguaua..	1	0	seq
....................auacccuguagauccgaau..	1	0	seq
....................auacccuguagauccgaauu...	5	0	seq
....................auacccuguagauccgaauuu..	35	0	seq
....................auacccuguagauccgaauuug...	102	0	seq
....................auacccuguagauccgaauuugu..	706	0	seq
....................auacccuguagauccgaauuugug...	69	0	seq
....................auacccuguagauccgaauuugugu..	12	0	seq
.....................uacccuguagauccgaau..	17	0	seq
.....................uacccuguagauccgaauu...	194	0	seq
.....................uacccuguagauccgaauuu..	993	0	seq
.....................uacccuguagauccgaauuug...	3047	0	seq
.....................uacccuguagauccgaauuugu..	107388	0	seq
.....................uacccuguagauccgaauuugug...	25001	0	seq
.....................uacccuguagauccgaauuugugu..	7654	0	seq
.....................uacccuguagauccgaauuugugua...	60	0	seq
.....................uacccuguagauccgaauuuguguaa..	6	0	seq
.....................uacccuguagauccgaauuuguguaagg..	2	0	seq
.....................uacccuguagauccgaauuuguguaaggaauuuu..	1	0	seq
......................acccuguagauccgaau..	1	0	seq
......................acccuguagauccgaauu...	29	0	seq
......................acccuguagauccgaauuu..	157	0	seq
......................acccuguagauccgaauuug...	386	0	seq
......................acccuguagauccgaauuugu..	11673	0	seq
......................acccuguagauccgaauuugug...	10597	0	seq
......................acccuguagauccgaauuugugu..	4757	0	seq
......................acccuguagauccgaauuugugua...	35	0	seq
......................acccuguagauccgaauuuguguaa..	3	0	seq
.......................cccuguagauccgaauuugu..	24	0	seq
.......................cccuguagauccgaauuugug...	34	0	seq
.......................cccuguagauccgaauuugugu..	22	0	seq
.......................cccuguagauccgaauuugugua...	1	0	seq
........................ccuguagauccgaauuu..	1	0	seq

oar-miR-10a

```
gaucugucugucuucuguauauacccuguagauccgaauuuguguaaggaauuuugugaucacaaauucguaucuaggggaauauguaguugacauaaacacuccgcuc
........................ccuguagauccgaauuugug.................................................................    2    0    seq
........................ccuguagauccgaauuuguguaa..............................................................    1    0    seq
.........................cuguagauccgaauuugugu................................................................    1    0    seq
...........................................................ucacaaauucguaucuagggga............................    1    0    seq
...........................................................ucacaaauucguaucuaggggaa...........................    1    0    seq
............................................................cacaaauucguaucuagggga............................    2    0    seq
............................................................cacaaauucguaucuaggggaa...........................    1    0    seq
............................................................cacaaauucguaucuaggggaau..........................    3    0    seq
.............................................................acaaauucguaucuaggggaa...........................    2    0    seq
.............................................................acaaauucguaucuaggggaau..........................    28   0    seq
..............................................................caaauucguaucuagggga............................    7    0    seq
..............................................................caaauucguaucuaggggaa...........................    17   0    seq
..............................................................caaauucguaucuaggggaau..........................    717  0    seq
..............................................................caaauucguaucuaggggaaua.........................    4    0    seq
..............................................................caaauucguaucuaggggaauau........................    4    0    seq
...............................................................aaauucguaucuagggga............................    1    0    seq
...............................................................aaauucguaucuaggggaa...........................    1    0    seq
...............................................................aaauucguaucuaggggaau..........................    97   0    seq
...............................................................aaauucguaucuaggggaaua.........................    8    0    seq
...............................................................aaauucguaucuaggggaauau........................    3    0    seq
................................................................aauucguaucuaggggaauau........................    7    0    seq
```
